# Fatal Early-Onset Aspergillosis in a Recipient Receiving Lungs From a Marijuana-Smoking Donor: A Word of Caution

**DOI:** 10.3389/ti.2022.10070

**Published:** 2022-02-14

**Authors:** Eleonora Faccioli, Federica Pezzuto, Andrea Dell’Amore, Francesca Lunardi, Chiara Giraudo, Marco Mammana, Marco Schiavon, Antonello Cirnelli, Monica Loy, Fiorella Calabrese, Federico Rea

**Affiliations:** ^1^ Thoracic Surgery and Lung Transplant Unit, Department of Cardiac, Thoracic, Vascular Sciences and Public Health, University of Padua, Padua, Italy; ^2^ Pathology Unit, Department of Cardiac, Thoracic, Vascular Sciences and Public Health, University of Padua, Padua, Italy; ^3^ Radiology Unit, Department of Medicine, University of Padua, Padua, Italy; ^4^ Forensic Pathology Center “CML”, Portogruaro, Italy

**Keywords:** lung transplantation, Aspergillosis, marijuana-smoking donor, infection, fatal outcome

Dear editors,

We are aware that donors tobacco smoking history is quite common in the lung donor pool and several studies have investigated this aspect in order to understand whether this habit may influence the outcomes of recipients transplanted with lungs from smoking-donors (1,2). At the same time, there is very little literature focusing on donors’ marijuana smoking history as a factor affecting lung transplant (LTx) outcomes with conflicting results on early and intermediate (3,4) lung transplant outcomes.

We would like to focus the attention of the clinicians involved in LTx on a case of a 50 years-old patient, affected by idiopathic pulmonary fibrosis in therapy with nintedanib, who underwent bilateral lung transplantation at our Unit.

The donor was a 21 year-old male patient, admitted to the Intensive Care Unit (ICU) for a traumatic brain hemorrhage, with an unremarkable medical history except for cannabis abuse. Oto Score was 0 and all microbiological tests were negative.

The lung transplantation was performed with the usual surgical technique and peri and post-operative antibacterial prophylaxis was performed with combined antibiotics.

Antifungal and Cytomegalovirus prophylaxis and immunosuppressive therapy were based on aerosolized amphotericin B, ganciclovir and corticosteroids, mycophenolate mofetil, and cyclosporine respectively.

During the post-surgical phase, one blood culture was positive for *Staphilococcus Epidermidis* and two bronchial aspirates were positive for *Acinetobacter baumannii* and *Klebsiella pneumonia*, respectively.

Since the clinical conditions of the recipient were progressively improving, he was considered ready to be discharged. Before discharge, he underwent a bronchoscopy to perform surveillance trans-bronchial biopsies. The sample was insufficient. The histological examination showed diffuse alveolar damage and organizing pneumonia, as signs of ischemia reperfusion injury, while neither acute cellular rejection/lymphocytic bronchiolitis, infection, or marijuana-related lesions were detected.

The day after the procedure, the recipient presented a massive hemoptysis with cardiac arrest that required re-intubation and re-admission to the ICU. Since then, numerous episodes of hemoptysis have occurred and the patient died 10 days later because of hypovolemic shock.

A CT scan, performed the day before the exitus, showed multiple bilateral nodules which have been due to the hemorrhagic episodes and a small wedge-shaped cavitated lesion (arrow) could suggest, ex post, a possible aspergillosis (Supplementary Figure 1). An autopsy was then performed and histological examination of the lungs revealed an invasive pulmonary aspergillosis (IPA) (Figure 1) and smoking-related lesions (chronic bronchiolitis/bronchitis with infiltration of heavily pigmented macrophages) in the few evaluable areas. A timeline describing all the events is represented in Supplementary Figure 2.

**FIGURE 1:**
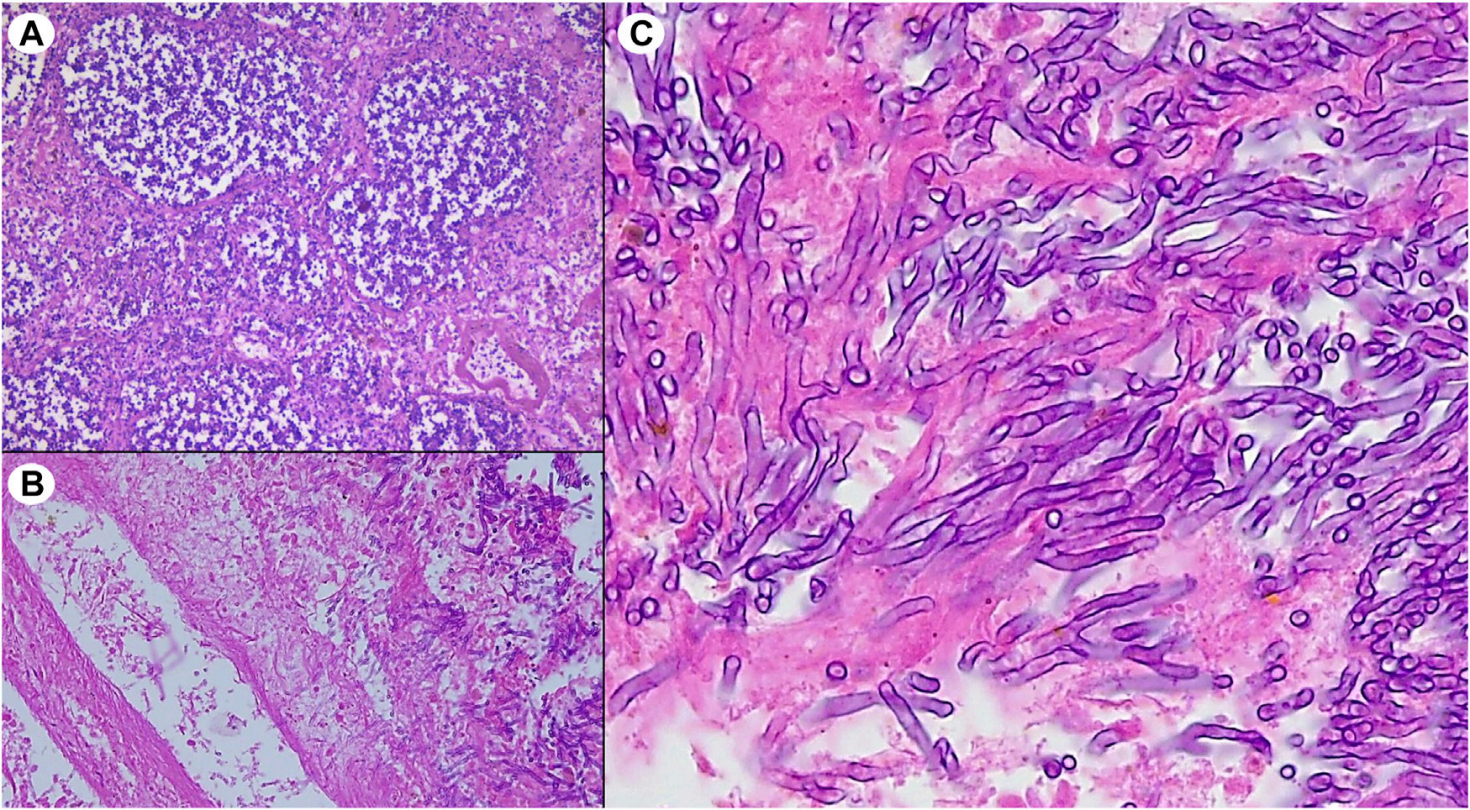
Histological lung sections from recipient’s autopsy showing **(A)** multiple foci of bronchopneumonia, **(B)** vascular erosion associated with widespread blood extravasation and, **(C)** well recognizable fungal branched hyphae compatible with *Aspergillus spp*.

A correlation between inhalation of marijuana and IPA has already been reported in renal (5) and bone marrow recipients (6) but, to the best of our knowledge, this is the first report of fatal early onset IPA in a patient who received lungs from a donor with ongoing marijuana use. We believe that, in our patient, a correlation between the donor’s marijuana smoking history and IPA could be supposed since no other explanation justified the development of such clinical picture.

However, it must be taken into account that such a clinical manifestation is anecdotal also considering the increasing prevalence of cannabis use between 2010 and 2019 in Europe (+27% in the population between 15 and 64 years) (7).

Despite this, since organ donors are often included in this age group, we would like to raise awarness in clinicians suggesting an accurate evaluation of the lungs retrieved from donors with ongoing marijuana abuse.

In case of young donors with cannabis smoking history, the pre-emptive research of fungi (especially Aspergillus) on biological samples should always be encouraged. At the same time, more sensitive tools, like polymerase chain reactions, could help in the early detection of Aspergillus in recipients with bleeding unrelated to the surgical procedure undergone.

## Data Availability

The raw data supporting the conclusion of this article will be made available by the authors, without undue reservation.

## Supplementary Material

Supplementary Figure 1 CT scan iperformed the day before the exitus

Supplementary Figure 2 timeline with representation of the events

## Abbreviations

ICU, intensive care unit; IPA, invasive pulmonary aspergillosis; LTx, lung transplantation

## References

[1] Schiavon M Lloret Madrid A Lunardi F Faccioli E Lorenzoni G Comacchio GM Short- and Long-Term Impact of Smoking Donors in Lung Transplantation: Clinical and Pathological Analysis. Jcm (2021). 10:2400. 10.3390/jcm10112400 34071675 PMC8199202

[2] Oto T Griffiths AP Levvey B Pilcher DV Whitford H Kotsimbos TC A Donor History of Smoking Affects Early but Not Late Outcome in Lung Transplantation. Transplantation (2004). 78:599–606. 10.1097/01.tp.0000131975.98323.13 15446321

[3] Okahara S Levvey B McDonald M D'Costa R Opdam H Pilcher DV Influence of the Donor History of Tobacco and Marijuana Smoking on Early and Intermediate Lung Transplant Outcomes. J Heart Lung Transplant (2020). 39:962–9. 10.1016/j.healun.2020.05.019 32593560

[4] Mohite PN Zeriouh M Sáez DG Popov A-F Sabashnikov A Zych B Influence of History of Cannabis Smoking in Selected Donors on the Outcomes of Lung Transplantation. Eur J Cardiothorac Surg (2017). 51:142–7. 10.1093/ejcts/ezw255 28077504

[5] Marks WH Florence L Lieberman J Chapman P Howard D Roberts P Successfully Treated Invasive Pulmonary Aspergillosis Associated with Smoking Marijuana in a Renal Transplant Recipient. Transplantation (1996). 61:1771–4. 10.1097/00007890-199606270-00018 8685958

[6] Hamadeh R Ardehali A Locksley RM York MK. Fatal Aspergillosis Associated with Smoking Contaminated Marijuana, in A Marrow Transplant Recipient. Chest (1988). 94:432–3. 10.1378/chest.94.2.432 3293934

[7] Manathey J Freeman TP Kilian K Lopez-Pelayo H Rehm J. Public Health Monitoring of Cannabis Use in Europe: Prevalence of Use, Cannabis Potency, and Treatment Rates. The Lancet Reg Health- Europe (2021). 10:100227. 10.1016/j.lanepe.2021.100227 PMC8589728 34806072

