# Supplementary figures and images for: Fatal Early-Onset Aspergillosis in a Recipient Receiving Lungs From a Marijuana-Smoking Donor: A Word of Caution
Source: Transpl Int. 2022 Feb 14;35:10070. doi: 10.3389/ti.2022.10070 (PMC8883434; doi:10.3389/ti.2022.10070)

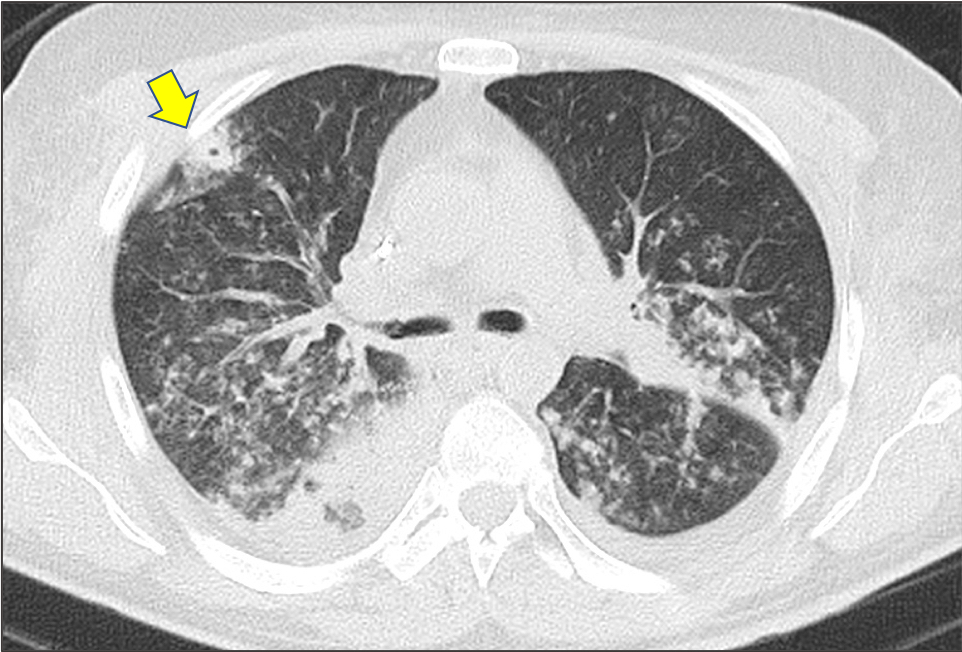

Supplement: Supplementary file 1 [file Image1.JPEG]

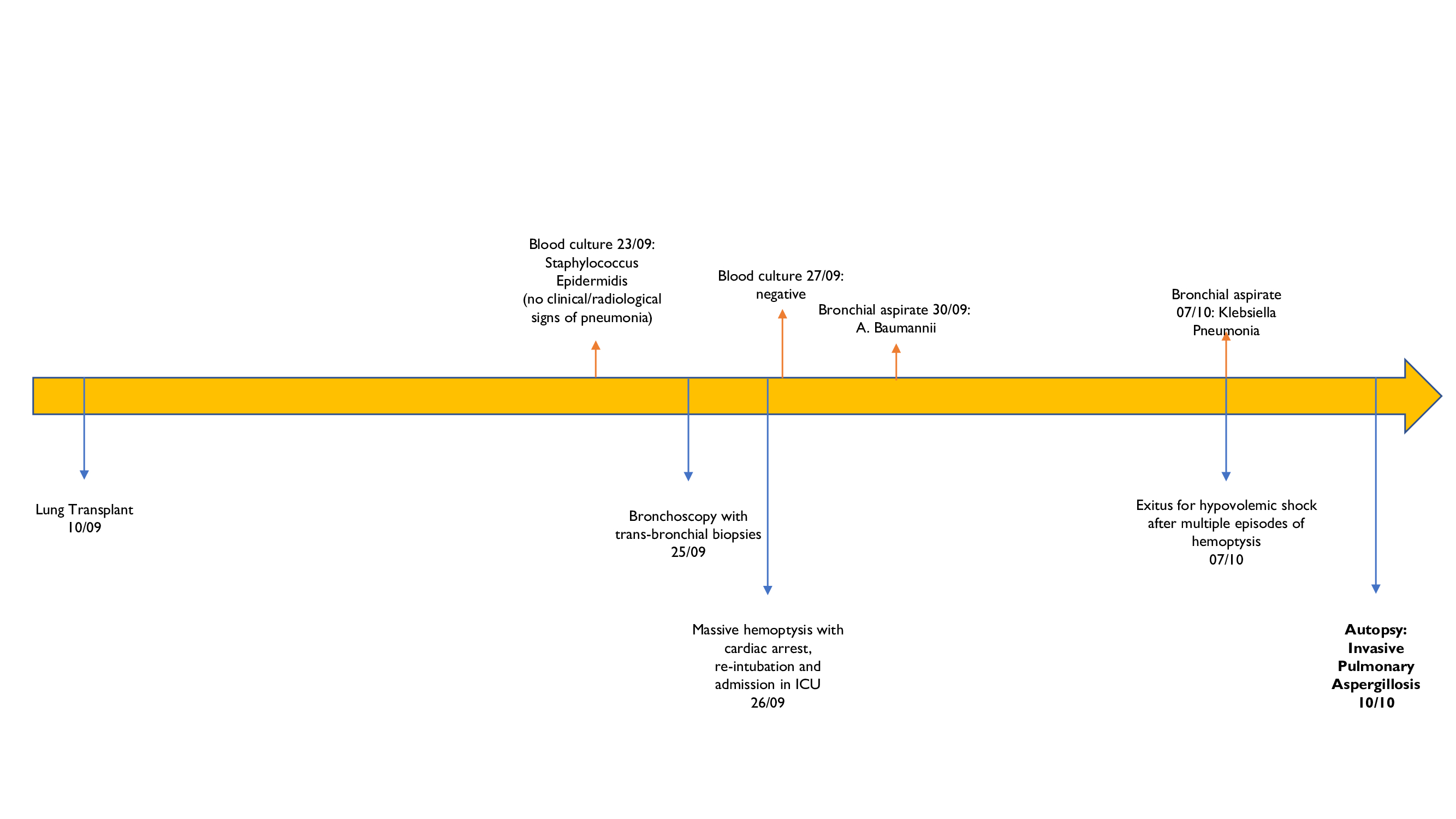

Supplement: Supplementary file 2 [file Image2.JPEG]
